# Primary ciliary dyskinesia complicated with diffuse panbronchiolitis: a case report and literature review

**DOI:** 10.1111/crj.12089

**Published:** 2014-01-01

**Authors:** Wei Chen, Changzhou Shao, Yuanlin Song, Chunxue Bai

**Affiliations:** 1 Shanghai Institute of Respiratory Diseases, Zhongshan Hospital, Shanghai Medical College of Fudan UniversityShanghai, China; 2 Department of Respiratory Medicine, Huai’an First People’s Hospital, Nanjing Medical UniversityHuai’an, China

**Keywords:** case report, clinical profiles, diffuse panbronchiolitis, literature review, primary ciliary dyskinesia

## Abstract

**Background:**

Fifty percent of patients with primary ciliary dyskinesia (PCD) have situs inversus. Diffuse panbronchiolitis (DPB) might be one of the characteristic features of the lung in PCD.

**Methods:**

We reported a case of PCD without situs inversus, yet complicated with DPB, and did literature review.

**Results:**

A 34-year-old nonsmoking Chinese woman with 6-year primary infertility suffered from recurrent episodes of respiratory tract infections since childhood. Lung auscultation revealed end-inspiratory coarse crackles. Pulmonary function tests demonstrated mild obstructive ventilation functional impairment. Lung biopsy showed respiratory bronchiolitis. Nasal mucosa cilia showed the absence of both outer and inner dynein arms of the microtubules. Saccharin test was positive. Chest images showed bronchiectasis and bronchiolitis but no situs inversus. Paranasal sinus computed tomography (CT) showed maxillary sinusitis and ethmoid sinusitis. A culture of bronchoalveolar lavage fluid was positive for *Pseudomonas aeruginosa*. Her conditions improved in clinical symptoms and CT images after 2 months of treatment with azithromycin. Literature review revealed that very rare patients were diagnosed as PCD complicated with diffuse DPB, and all of them had situs inversus.

**Conclusions:**

The association of DPB might be one of the characteristic features of the lung in PCD. Further studies on the concurrence of these two diseases are suggested so as to elucidate the mechanism of both.

Please cite this paper as: Chen W, Shao C, Song Y and Bai C. Primary ciliary dyskinesia complicated with diffuse panbronchiolitis: a case report and literature review. Clin Respir J 2014; 8: 425–430.

## Introduction

Primary ciliary dyskinesia (PCD) is usually an autosomal recessive disease characterized by chronic upper and lower respiratory tract infection, and in nearly 50% cases, mirror image arrangement 1. As suggested by studies, diffuse bronchiolitis might be one of the characteristic features of the lung in PCD 2. Diffuse panbronchiolitis (DPB) is an idiopathic inflammatory disease, well recognized in Japan and principally affecting the respiratory bronchioles, causing a progressive suppurative and severe obstructive respiratory disorder 3. Here we report a case of a 34-year-old woman diagnosed as PCD complicated with DPB. This appears to be the first report of PCD without situs inversus, yet complicated with DPB.

## Case report

### General medical history

A 34-year-old nonsmoking Chinese woman with chronic cough and a 6-year history of primary infertility was transferred to the Department of Pulmonary Medicine (Zhongshan Hospital, Fudan University, China) as she had been experiencing mild dyspnea on exertion, intermittent nasal congestion for half a year, a productive cough and purulent sputum for 2 months. Her parents were nonconsanguineous. The patient suffered from recurrent episodes of respiratory tract infections since her childhood. She had previously been diagnosed as chronic sinusitis and bronchiectasis.

### Physical examination

The patient’s vital signs were as follows: temperature, 36.5 C; respiratory rate, 20 breaths/min; blood pressure, 120/70 mmHg; and heart rate, 86 beats/min. Chest examination revealed end-inspiratory coarse crackles. She appeared with minimal clubbing figures.

### Accessory examination

**Figure 1:**
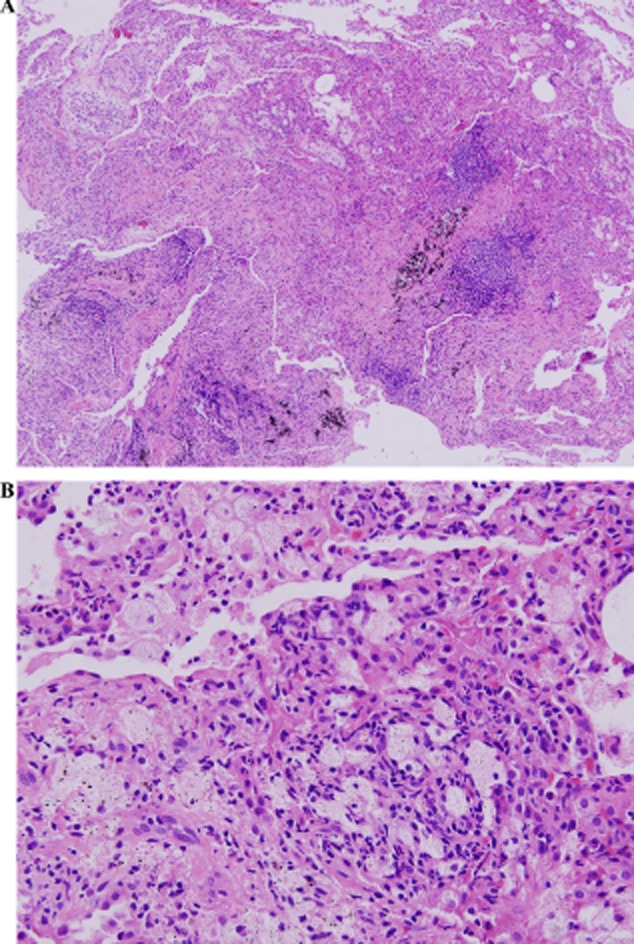
Pathology change of transbronchial lung biopsy (hematoxylin and eosin staining, A × 40; B × 200) showed fibrous tissue hyperplasia, inflammatory cell infiltration and histiocytic reaction in alveolar space. Chronic inflammatory cell infiltration and foamy phagocytes groups in small wall-thickened bronchioles and interstitial lung.

**Figure 2:**
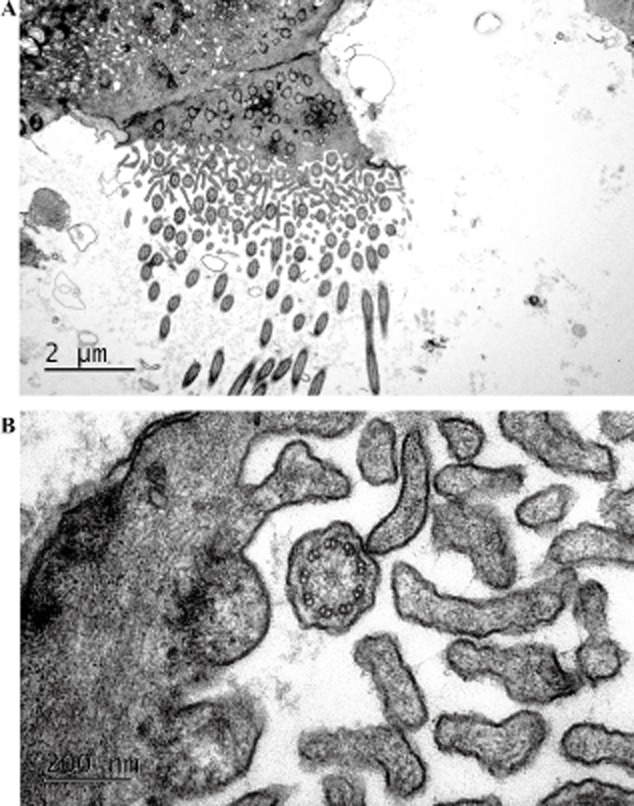
Electron microscopy of nasal mucosa showed cilia with the absence of both outer and inner dynein arms of the microtubules (transmission electron microscopy, 3 200 000).

Routine blood tests were unremarkable. Other laboratory findings included the following: erythrocyte sedimentation rate, 23 mm/h; C-reactive protein, 26 mg/L; and CD4/CD8, 0.6. The serum cold hemagglutinin testing, auto-antibodies and serum total immunoglobulin E were all negative. Sputum cultures isolated *Pseudomonas aeruginosa*. Pulmonary function tests were as follows: vital capacity, 3.3 L (76% predicted); forced vital capacity (FVC), 3.84 L (84.1% predicted); forced expiratory volume at 1 s (FEV_1_), 2.76 L (72% predicted); FEV_1_/FVC ratio, 64.5; and diffusion capacity of the lung for carbon monoxide, 30 mL/min/mmHg (96% predicted). Arterial blood gas levels on room air included the following: pH, 7.44; PaCO_2_, 32 mm Hg; and PaO_2_, 80 mmHg. Bronchoscopy showed massive amount of yellowish bronchial secretions from the right middle lobe bronchi. *P. aeruginosa* was also isolated from bronchoalveolar lavage fluid. Drug sensitivity tests were as follows: amikacin, S; ceftazidime, S; imipenem (Tienam, Merck & Co., Inc., Whitehouse Station, NJ, USA), S; cefepime, I; piperacillin/tazobactam, R; cefoperazone/sulbactam, R; levofloxacin, S; aztreonam, S; polymyxin B, S; and piperacillin, R (S, susceptible; I, intermediate; and R, resistance). The pathology change of transbronchial lung biopsy showed respiratory bronchiolitis (Fig. 1). A saccharin test demonstrated a mucociliary transport time of 40 min. After 20 days of combination therapy with ceftazidime and azithromycin, no pathogens can be found in sputum cultures. Then the absence of both outer and inner dynein arms of the microtubules was found by examination of the nasal mucosal biopsy specimen under transmission electron microscopy (Fig. 2).

### Radiographic findings

**Figure 3:**
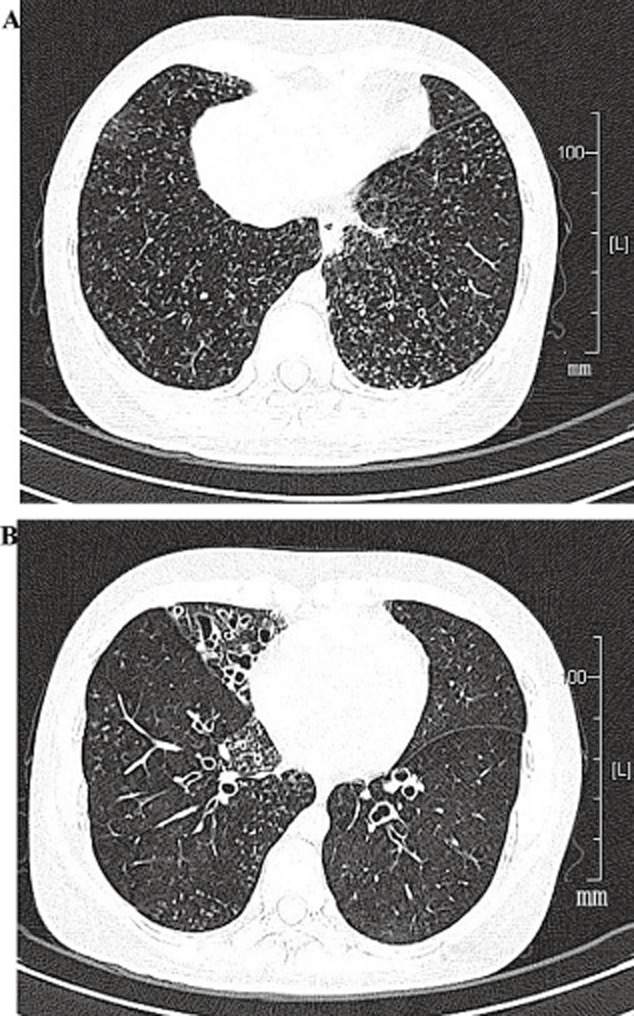
Computed tomography (CT) images of the chest. (A) High-resolution computed tomography (HRCT) shows bronchiectasis and bronchiolitis, ring-shaped or ductal opacities in upper, middle and lower lungs, some of which are accompanied by small nodules. (B) Bronchiectasis involved in right middle lobe.

**Figure 4:**
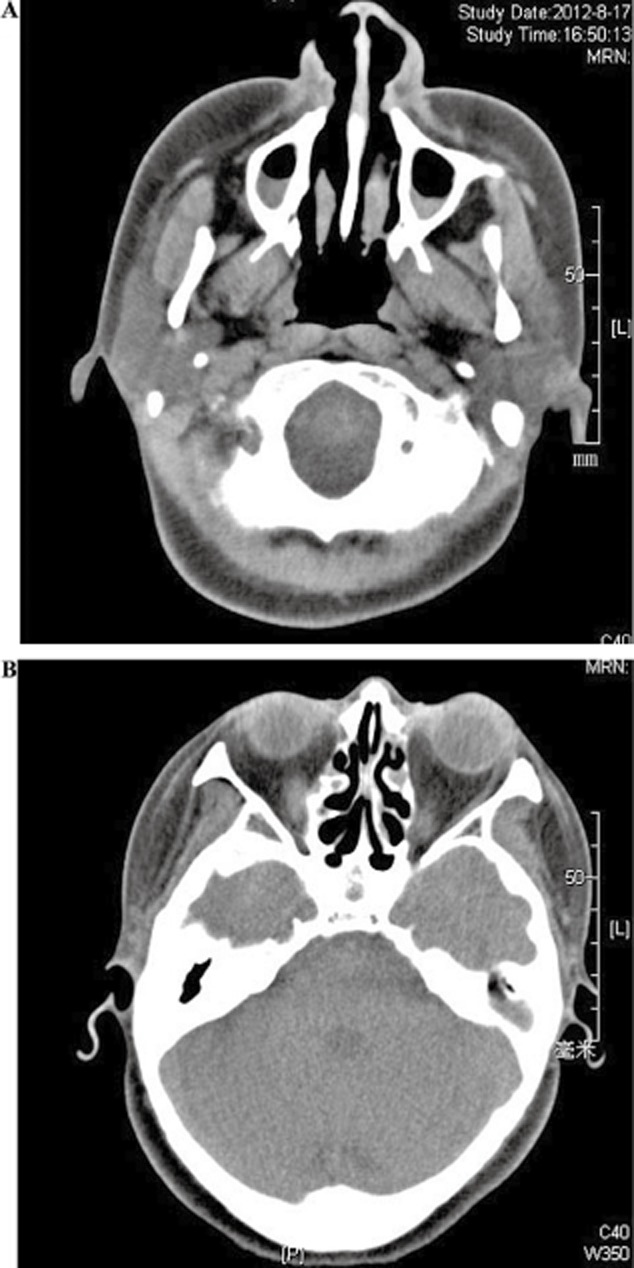
Computed tomography (CT) images of the sinuses showed chronic maxillary sinusitis and (B) ethmoid sinusitis.

High-resolution computed tomography (HRCT) showed bronchiectasis and bronchiolitis, ring-shaped or ductal opacities throughout both lungs, and some of which were accompanied by small nodules. Bronchiectasis mainly involved in the right middle lobe (Fig. 3). Paranasal sinus CT showed chronic maxillary sinusitis and ethmoid sinusitis (Fig. 4).

### Diagnosis and treatment

**Figure 5:**
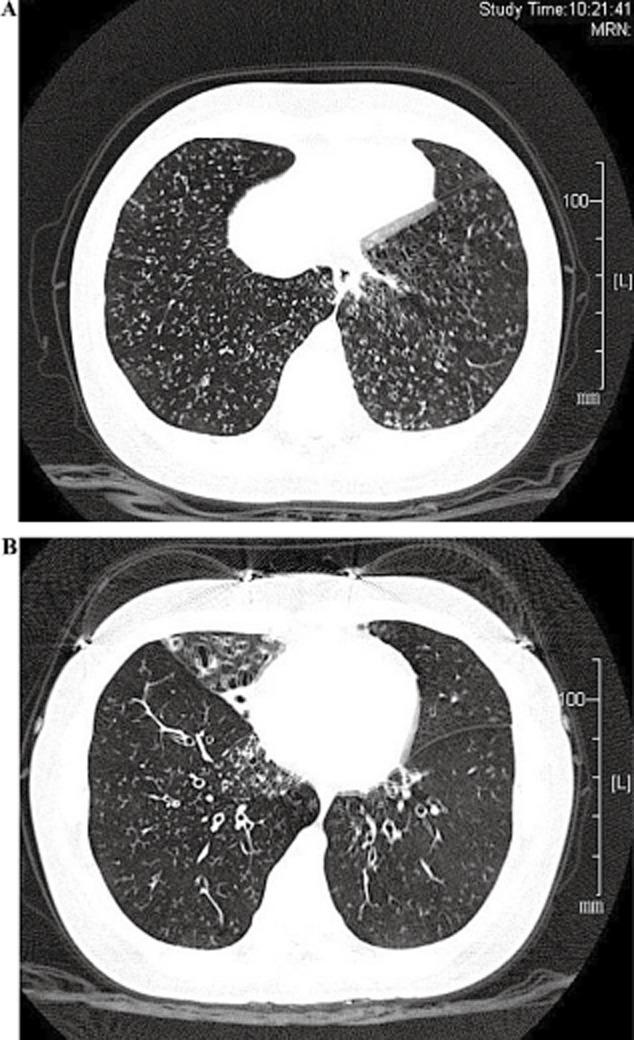
High-resolution computed tomography (HRCT) images after 20 days of azithromycin therapy. (A) Nodular shadows were obviously attenuated, (B) but bronchiectasis did not change.

Based on the clinical profiles above, the patient was diagnosed as PCD complicated with DPB. The initial treatment protocol was combination therapy with ceftazidime and azithromycin. Azithromycin (500 mg, once a day) was administrated intravenously in the first 5 days and then taken orally (500 mg, once a day) as her symptoms improved. Adjuvant therapy included inhaled salmeterol/fluticasone in order to relieve the nasal symptoms and dyspnea. After 20 days of treatment, the presenting symptoms of dyspnea on exertion, cough and nasal congestion improved or stabilized in this patient. The sputum volume reduced significantly, and the sputum color changed from purulence to phlegm; the result of sputum cultures became negative. Chest CT showed that nodular shadows were mildly attenuated after 2 months of azithromycin therapy, but bronchiectasis was not improved (Fig. 5). Pulmonary function tests were reviewed after 2 months. It showed that FEV_1_ and FVC were improved mildly for the inadequate treatment. The results were as follows: FVC, 4.02 L (88.2% predicted); FEV_1_, 2.98 L (78% predicted); and FEV_1_/FVC ratio, 70.5. Arterial blood gas suggested that PaO_2_ increased from 80 mmHg to 90 mmHg. The result of sputum cultures was negative this time.

## Discussion

PCD is a rare autosomal recessive disease 4. At least 50% of patients may present with unexplained neonatal respiratory distress ranging from supplementary oxygen to ventilator support in severe cases 5. The most common symptoms include bronchiectasis in respiratory, situs inversus in cardiovascular, male infertility due to impaired sperm motility and female ectopic pregnancy or infertility because of ciliary dysfunction in the fallopian tubes 1. Characterized by the triad of situs inversus, bronchiectasis and chronic sinusitis, Kartagener syndrome (KS) is the first reported PCD 1. Only half of the patients affected by PCD present all of the symptoms, a condition designed complete KS, compared with incomplete KS, typically defined as cases in which situs inversus does not occur 6. The most popular screening test for PCD is the saccharin test, and the specific diagnosis requires examination of cilia by light and electron microscopy 7. The reported prevalence ranges from 1/2000 to 1/40 000 in the general population 8, but the incidence of PCD in Chinese is unknown. In the review of the literature, only 182 cases of PCD were reported in China 9. Among the 182 cases, only 10 patients were not accompanied by situs inversus. Therefore, PCD without situs inversus was often missed by the clinician. The patient in this report suffered from recurrent episodes of respiratory tract infections since her childhood. Her clinical manifestations included chronic sinusitis, primary infertility, bronchiectasis and bronchiolitis, but no situs inversus in HRCT images. The saccharin test was positive. Electron microscopy of nasal mucosa showed cilia with the absence of both outer and inner dynein arms of the microtubules. All of these findings were consistent with the features of PCD.

Diagnostic criteria for DPB proposed in 1998 10 by a working group of the Ministry of Health and Welfare of Japan are as follows:

- persistent cough, sputum and exertional dyspnea;
- history of or current chronic sinusitis;
- bilateral diffuse small nodular shadows on a plain chest X-ray film or centrilobular micronodules on chest CT images;
- coarse crackles;
- FEV_1_/FVC less than 70% and PaO_2_ less than 80 mmHg;
- titer of cold hemagglutinin equal to or higher than 64.

Definite cases should fulfill the first three criteria mentioned above and at least two other remaining criteria.

Histologically, DPB is characterized by chronic inflammation, localized mainly in the respiratory bronchioles and adjacent centrilobular regions, with characteristic interstitial accumulation of foamy histiocytes, neutrophils and lymphocyte infiltration. Neutrophils and T lymphocytes, particularly CD8+ cells, together with the cytokines interleukin-8 and macrophage inflammatory protein-1, are believed to play key roles in the development of DPB 3. The prominent involvement of respiratory bronchioles is a distinctive feature of DPB; as in other forms of obliterative bronchiolitis, the main involved structures are membranous bronchioles 11. In advanced DPB, superinfection of *P. aeruginosa* reduces the lungs’ capacity for gas exchange, which brings about the progression of hypoxemia and, later, hypercapnia. Left untreated, the condition of patient with DPB deteriorates more rapidly than other chronic lung conditions, and the outcome is fatal 12. The patient in this report had a history of chronic cough and chronic sinusitis for more than 20 years and a 6-year history of primary infertility. Lung auscultation revealed end-inspiratory coarse crackles. Pulmonary function tests demonstrated mild obstructive ventilation functional impairment. HRCT findings were the presence of bronchiectasis and bronchiolitis. The result of bronchoalveolar lavage fluid cultures was *P. aeruginosa*. These features and pathological changes were all consistent with the diagnostic criteria of DPB.

The association of PCD in patients with DPB is exceedingly rare. In review of the literature, only 17 cases of KS complicated with DPB were reported since 1999, most in East Asians, including nine cases in China 2,13–16. But all of these cases had situs inversus. PCD without situs inversus complicated with DPB in patients has not been reported previously. Amitani *et al*. 17 reported nine cases of PCD in 1990. The clinical diagnoses in their report included six cases of KS, two cases of DPB and one case of bronchiectasis. They described diffuse micronodular shadows on the chest radiograph in one patient with KS and in both patients with DPB. However, DPB in their report was a clinical diagnosis and lacked histopathological analysis. Although the etiology of DPB is as yet unknown, they also suggested that one of the etiological factors of DPB might be PCD. There were striking similarities between PCD and DPB in clinical features, radiography images and pulmonary function tests. Although the histopathological features showed that the primary inflammatory lesions were in the membranous bronchioli in KS and in the respiratory bronchioli in DPB 13, the lesions were all located in bronchioli. Homma *et al*. 13 also demonstrated that the diffuse centrilobular small nodules on the chest CT images mainly corresponded to membranous bronchiolitis.

PCD is genetically heterogeneous, being caused by mutations in a number of different genes 18–20. The DNAH5 gene and the DNAI1 gene, two genes encoding dyneins, have been confirmed to have a significant correlation with PCD. DPB is not a simple genetic disorder, but is considered to be a multifactorial disease occurring in adulthood. Human leukocyte antigen (HLA)-B54, known as an ethnic antigen unique to East Asians, was strongly associated with the disease in Japan 21, and the HLA-A11 antigen in Korea 22 and China 23. Although many loci have been mapped in PCD and DPB, the causative gene remains to be determined. But it is supposed that there may be some association in genetic mutation between PCD and DPB.

Recurring lung infection is the most common clinical feature occurred in both PCD and DPB. PCD lung disease and DPB are progressive with no adequate treatment, and early therapeutic interventions result in better symptom control. The common infecting organisms in these two diseases are *Haemophilus influenza* and *P. aeruginosa* 3,7. The advent of macrolide therapy has strikingly changed the prognosis of DPB, notwithstanding that we still do not know the exact mechanism of this disease. From the clinical experiences of the efficacy of macrolides in patients with DPB, long-term treatment with macrolides has been used for patients with PCD in Japan 24. And the authors suggested that macrolides may have the potential to ameliorate the natural course of PCD with mild ciliary dysfunction via the modification of the activities of the immune system.

In conclusion, this report describes a case of a 34-year-old woman with chronic cough and infertility diagnosed with PCD complicated with DPB. Although the exact mechanism and relationship of these two diseases are still unclear, it is suggested that DPB might be one of the characteristic features of the lung in PCD. Further studies on the concurrence of these two diseases are suggested so as to elucidate the mechanism of both.
